# First completely robot-assisted retroperitoneal nephroureterectomy with bladder cuff: a step-by-step technique

**DOI:** 10.1007/s00345-021-03920-1

**Published:** 2022-01-17

**Authors:** P. Sparwasser, S. Epple, A. Thomas, R. Dotzauer, K. Boehm, M. P. Brandt, R. Mager, H. Borgmann, M. M. Kamal, M. Kurosch, T. Höfner, A. Haferkamp, I. Tsaur

**Affiliations:** Department of Urology, University Medical Center Johannes Gutenberg University, Langenbeckstr. 1, 55131 Mainz, Germany

**Keywords:** Nephroureterectomy, Retroperitoneal, Robotic surgery, DaVinci, Trocar placement, Bladder cuff

## Abstract

**Introduction:**

While various surgical techniques have been reported for open and minimally invasive treatment of upper tract urothelial cancer (UTUC), the procedure of robot-assisted nephroureterectomy (NU) with bladder cuff has never been reported using only retroperitoneum without entering abdominal cavity. We developed a novel port placement and technique allowing to perform robot-assisted NU by a unique retroperitoneal approach.

**Methods:**

Between February and June 2021 patients with history of UTUC were treated by robot-assisted NU completely restricted to retroperitoneal space using a singular trocar placement and a two-step docking without relocation of the surgical robot. Patient characteristics, perioperative outcomes and short-term follow-up were prospectively analyzed.

**Results:**

The analysis included five patients [median age: 73 years; BMI: 27.2 kg/m^2^; Charlson comorbidity index 5]. All five patients had UTUC with a mean tumor size of 3.02 cm (range 0.9–6.0). UTUC was localized to distal ureter in two and to kidney in three cases. No positive surgical margins were noted for all patients with UTUC [1 low-grade and 4 high-grade]. Retroperitoneal lymphadenectomy in three patients did not reveal positive nodes. No intraoperative adverse events exceeding EAUiaiC classification ≥ 2 were observed, while median EBL was 150 ml (IQR 100–250). No patient experienced postoperative complications exceeding Clavien–Dindo classification ≥ 3a. Median hospital stay was 5.4d without any 30-d readmission.

**Conclusion:**

We demonstrate safety and feasibility of the first entire robot-assisted retroperitoneal nephroureterectomy (RRNU) with bladder cuff. This surgical technique is easily reproducible, while surgical outcomes are similar to other established techniques.

**Supplementary Information:**

The online version contains supplementary material available at 10.1007/s00345-021-03920-1.

## Introduction

Up to 5–10% of urothelial carcinoma become manifest in the upper urothelial tract, whereas its incidence increases continuously due to demographic change [1]. The main aspect favoring radical surgery is that almost two-thirds of UTUC are detected in an invasive stage [1]. Thus, NU currently represents standard care for the most cases of UTUC [1]. Despite laparoscopic NU has been already introduced in 1991, many centers still perform open surgery, not least because of sophisticated utilization of laparoscopic instruments and a flat learning curve particularly for the bladder cuff [2]. With a growing adoption of robot-assisted surgery, NU is nowadays increasingly performed using this platform. Several approaches for robot-assisted NU have been reported so far. The most common way to perform this surgery is currently transperitoneal approach, for which several port placements have been reported [3–5]. In this context several colleagues [6–8] demonstrated a feasible access to the upper abdomen (nephrectomy portion) and simultaneously to the lower abdomen (bladder cuff excision) using DaVinci^R^ robotic platform for a single-docking technique. While Patel and colleagues proposed their port placement in a straight line to the linea semilunaris lateral to the rectus abdominis muscle [6] quite similar to Zargar et al. [7], Darwiche et al. set up an oblique line for port placement beginning from subcostal space and ending near to linea alba in the lower abdomen [8]. Importantly, transperitoneal approaches necessitate mobilization of the bowel by paracolic incision of the dorsal peritoneum to access retroperitoneal space. Based on the experience from robot-assisted partial nephrectomy a strictly retroperitoneal approach might in turn be beneficial for the control of hilar structures and reduction of intraoperative blood loss, operative time and hospital stay by decreasing postoperative discomfort especially triggered by pain and intestinal atony [9–11]. Moreover, retroperitoneal approach might be preferential for patients with previous abdominal surgery to avoid intraperitoneal adhesions and occasionally time-consuming adhesiolysis [9]. To date, a number of studies described retroperitoneal access for robot-assisted NU, but none of these surgeries were completed under robotic assistance [4, 12, 13]. In the majority of cases management of the bladder cuff after robot-assisted retroperitoneal nephrectomy was only possible through intraoperative switch to either conventional laparoscopic surgery or even open surgery [4, 12, 13].

We aimed at assessing our innovative surgical approach with a singular trocar placement for the first RRNU in which all surgical steps are completed using the DaVinci^R^Xi robotic platform.

## Methods

### Study population

Between February and August 2021 five consecutive patients diagnosed with a nonmetastatic UTUC were subjected to RRNU. All surgical procedures of the newly developed technique were carried out by the same operation team (console surgeon: I.T., assistant surgeon: P.S.). Lymphadenectomy was performed in case of radiologically suspicious regional lymph nodes. All patients signed an informed consent. In all cases, ureteral stent was in situ during robotic procedure either following previous ureteral biopsy through ureterorenoscopy or hydronephrosis.

### Surgical technique

#### Patient position

**Fig. 1:**
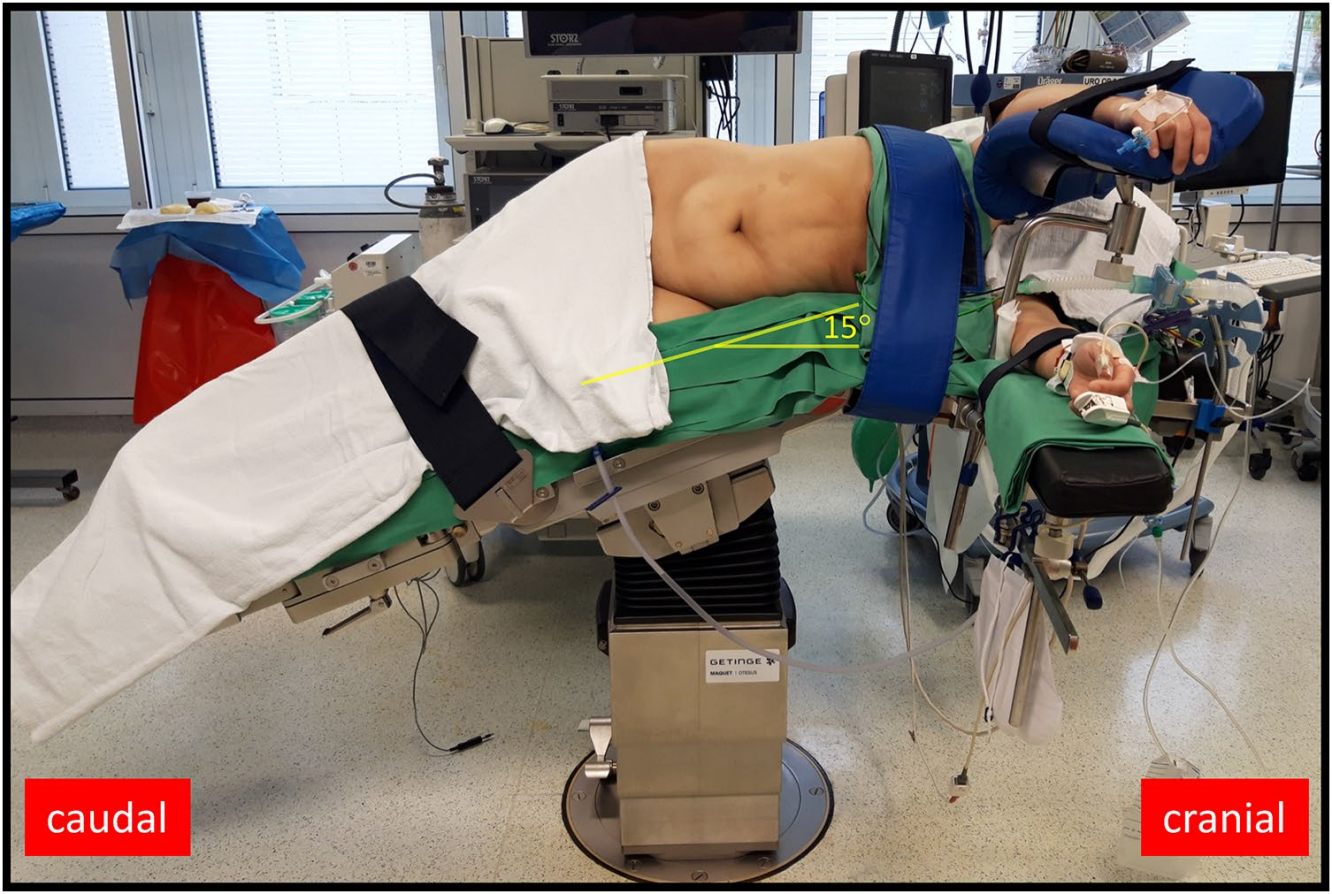
Patient position. The patient is placed in a 90° flank position with moderate table flexion (15°) and both arms were positioned perpendicular to the torso

The position on the operative table is comparable to that of the robot-assisted retroperitoneal partial nephrectomy [8]. The patient is placed thereby in a 90° full flank position (Fig. 1). We induce a moderate table flexion of maximum 15° to the torso. Both arms are positioned perpendicular to the body to enable retroperitoneal trocar placement.

#### Port placement and docking

**Fig. 2:**
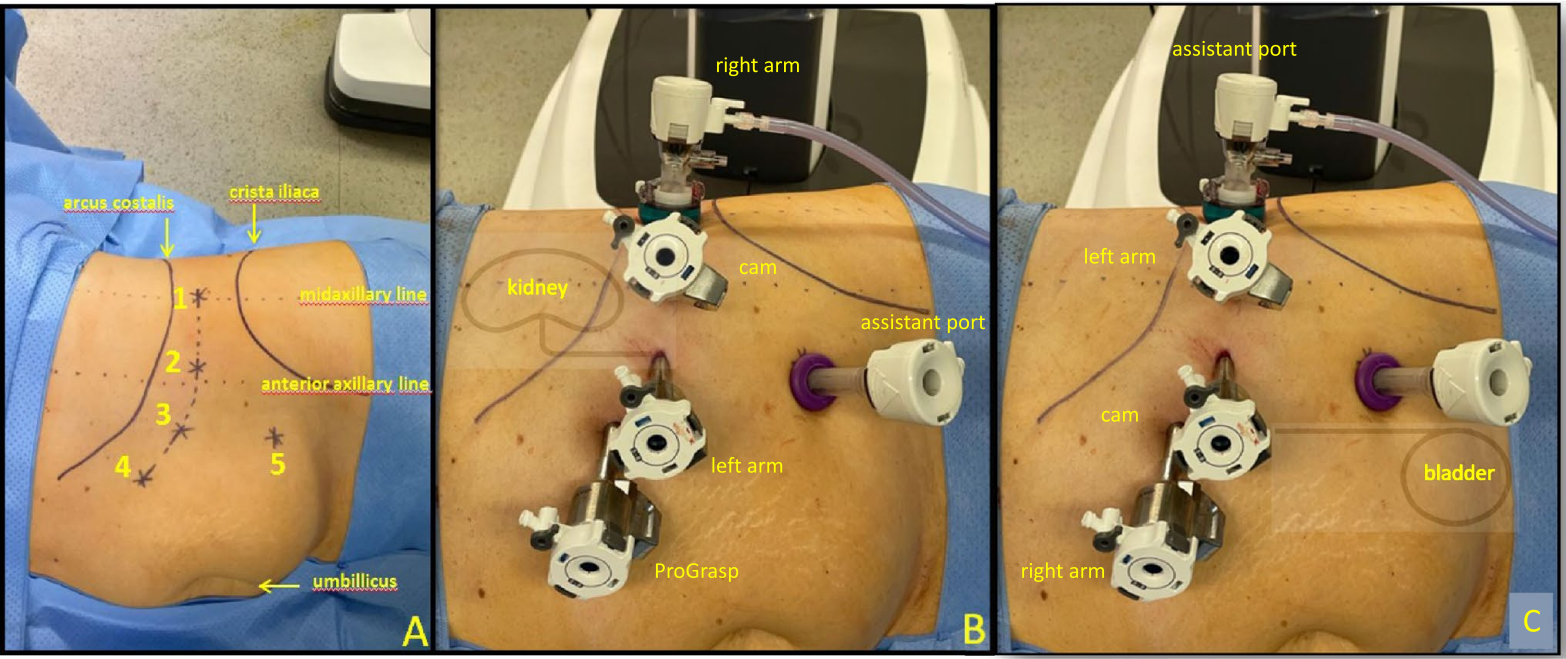
Trocar placement for nephrectomy portion and management of the bladder cuff. **A** Illustration of port arrangement with Hasson trocar (Nr.1), standard 8-mm DaVinci^R^-trocar (Nr.2 + 3 + 4) and 12-mm assistant port (Nr.5). **B** Trocar placement for four-arm configuration for nephrectomy portion with camera view towards cranial. **C** Trocar placement for three-arm configuration for dissection of the bladder cuff with camera view towards caudal after re-docking and 180° turnaround of the main robotic joint

A modified trocar placement for four arms using the DaVinci^R^Xi robotic platform (Intuitive^R^, Sunnyvale, USA) is performed (Fig. 2). The first incision is made approximately 1–2 fingerbreadths above the iliac crest in the triangle of Petit to enter retroperitoneal space through the lumbo-dorsal fascia. Hereafter the ballon-dilatator (KiiDissectingBALLON, AppliedMedical^R^, Rancho, USA) is applied to create retroperitoneal space under vision using a 0° camera for all surgical steps. The balloon-dilatator is replaced by a 12-mm Hasson trocar (KiiBALLON, AppliedMedical^R^, Rancho, USA), in which a standard 8-mm trocar (Intuitive^R^) is inserted. After insufflation of carbon dioxide a second 8-mm trocar is placed in the anterior axillary line under keeping a minimum distance of 6 cm between all ports. We used 12 mmHg continuously during the procedure. Peritoneum is now maximally medialized laparoscopically under vision and two additional ports are placed medially in a curved line parallel to the arcus costalis in addition to one 12-mm assistant port in the lower abdomen. The robot is now docked parallel to the spine with the trajectory of the arms towards the head. After finishing nephrectomy and release of the middle ureter, re-docking is performed by 180°-twist of the main joint of the robot without the need for relocation. Now the robot is docked in a three robotic-arm configuration with the trajectory of the arms towards the legs to enable dissection of the distal ureter and preparation of the bladder cuff. After re-docking the Hasson trocar serves now as assistant port for patient-side surgeon while the three medial trocars are connected to the robot.

#### Nephrectomy

**Fig. 3:**
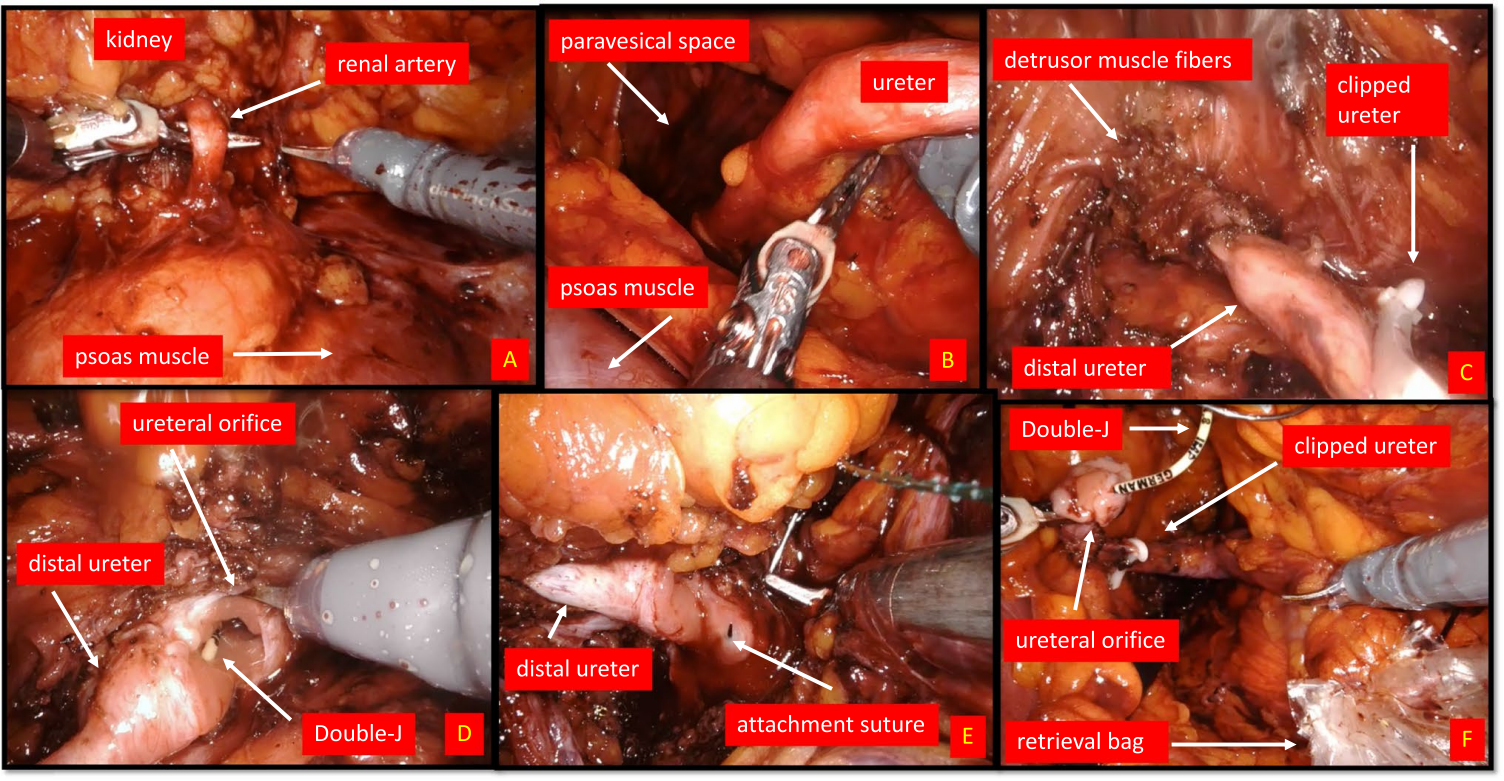
Intraoperative Surgical steps for left side RRNU. **A** Preparation of renal artery from dorsal before clipping using a clip applier (Hem-o-lok Teleflex^R^) for nephrectomy portion. **B** Dissection of the middle ureter with view to paravesical space after nephrectomy (the kidney is already mobilized towards cranial) and after re-docking DaVinci Xi^R^. The proximal and middle ureter were released by sharp and blunt dissection. **C** View at the bladder wall with step-by-step dissection of detrusor muscle fibers along the prevesical ureter. The distal ureter has been already clipped below the tumor using a Hem-o-lok clip. **D** Management of bladder cuff with excision of detrusor muscle till urothelial mucosa is exposed. Previous inserted Double-J is exposed after opening the bladder wall. **E** Before complete dissection of the bladder cuff an attachment suture (V-Loc 3-0; Covidien^R^) is placed to medial margin of ureteral orifice to prevent retraction of the bladder. **F** View at the completed bladder cuff after closure of the bladder defect using the attachment suture (V-Loc 3-0; Covidien^R^). The clipped ureter including the Double-J is being inserted in a retrieval bag

To control renal vessels, the kidney is elevated to the abdominal wall ventro-medially with the ProGrasp Forceps (Intuitive^R^) and the renal hilum is exposed (Fig. 3; video presentation). Once the hilar anatomy is clearly defined we used multiple Hem-o-lok clips (Teleflex^R^, Morrisville, USA) to seal the vessels prior to their division. Now we used sharp and blunt dissection for mobilization of the kidney. After releasing superior and lateral attachments of the kidney the kidney is fully freed and lies mobile in the renal fossa. Preparation of the ureter is thereby executed by following the ureter on the psoas muscle from proximal till its middle section. This corresponds to the maximum of the functional articulation for the DaVinci^R^Xi robot and re-docking is now necessary. After finishing preparation of the ureter we clipped the ureter to prevent cell spreading.

#### Management of bladder cuff

After re-docking (Fig. 2, video presentation) we released the ureter, still connected to the kidney, with blunt and sharp dissection and followed it downwards to its bladder orifice. During this step the patient-side surgeon medialized peritoneum with the grasping forceps. To identify exact boarders of the ureteral orifice the bladder is filled by saline through the catheter. The detrusor muscle is then dissected until bladder mucosa can be visualized and circumferential en-bloc excision was initiated. Before entire dissection of the bladder cuff is executed one suture V-Loc 3-0 (Covidien^R^, Dublin, Ireland) is attached at the medial dissection margin to prevent retraction of the bladder wall (Fig. 3, video presentation). We then resected the bladder cuff completely and bladder defect was closed in a running fashion using the attached suture. Patency check was warranted by irrigating the bladder catheter with saline.

#### Lymphadenectomy

Retroperitoneal lymph node dissection was performed selectively in patients according to the EAU Guidelines [1]. Lymphatic tissue was resected from renal hilum till the iliac vessels. Bigger lymphatics were sealed by Hem-o-lok clips.

#### Specimen extraction and closure

A laparoscopic entrapment sac (Inzii^R^12/15 mm, AppliedMedical^R^, Rancho, USA) is introduced by lower 12-mm assistant port and removed through the midaxillary incision after manual reposition. A 20 French Robinson drain (Braun^R^, Melsungen, Germany) is placed through lower 12-mm assistant port and the lumbo-dorsal fascia is closed with a running suture.

### Data analysis

Clinical data were collected in a dedicated database including patients age, body mass index (BMI), sex, Charlson Comorbidity Index (CIC), American Society of Anesthesiologists score (ASA), tumor size, estimated blood loss (EBL), length of hospital stay, transfusion rate, tumor stage, positive margins, pathologic data, as well as the number of intraoperative complications (EAUiaiC) [14] and postoperative complications (Clavien–Dindo) [15]. In addition time intervals of all surgical steps were recorded. Descriptive statistics were used, whereat we presented means for continuous variables and frequencies and proportions for categorical variables.

## Results

### Demographics

Five patients were treated with RRNU at our institution (Table 1). Median age was 73 years with median BMI of 27.2 kg/m^2^ and according to CIC, ASA and ECOG with a moderate health status on average. Previous operations included cholecystectomy, appendectomy, hysterectomy and prostatectomy.

**Table 1:** Demographics and pathology findings

Characteristics	Results
Patients, no	5
Median Age, year. (IQR)	73 (70–75)
Female sex, no. (s%)	3 (66,6)
Median BMI, kg/m^2^, (IQR)	27.2 (27.5–29.4)
ASA score ≥ 3, no. (%)	2 (40)
Median CCI score, (IQR)	5 (5–6)
Side, no	
Right	2
Left	3
Lymph node dissection, no. (%)	3 (60)
EAUiaiC (intraoperative complications) ≥ 2, no. (%)	1 (20)
Clavien–Dindo grade (postoperative complications) ≥ 3a, no. (%)	1 (20)
Median EBL, ml (IQR)	150 (100–250)
Drain removal postoperative, d (range)	3 (2–4)
Creatinine in drain fluid, no. (%)	0 (0)
Blood transfusion, no. (%)	1 (20)
Catheter removal, d (range)	5.4 (5–7)
Sufficient cystography, no. (%)	5 (100)
Hospital stay, d (range)	5,4 (5–7)
30-d readmission, no. (%)	0 (0)
Histology, no. (%)	
UTUC-Ta	2 (40)
UTUC-T1	2 (40)
UTUC-T3	1 (20)
Size UTUC, cm (range)	3.02 (0.9)
Location UTUC, no. (%)	
Kidney/proximal ureter	3 (60)
Mid/distal ureter	2 (40)
Grade, no. (%)	
High	4 (80)
Low	1 (20)
Positive surgical margin	0 (0)
LN status, no	
Positive LN, no. (%)	0 (0)
Negative LN, no. (%)	3 (100)
Follow-up, mo. (range)	6 (2–8)
Localized recurrence bladder, no. (%)	1 (20)
Systemic recurrence, no. (%)	0 (0)

ASA: American Society of Anesthesiologists; BMI: body mass index; EAUiaiC: Intraoperative Adverse Incident Classification by European Association of Urology; CCI: Charlson Comorbidity Index; EBL: Estimated Blood Loss; UTUC: upper urinary tract cancer; LN: lymph node IQR: interquartile range

### Surgical characteristics

Mean operative time was 189.2 min (Table 2). Time for the trocar placement including primary docking was 28 min and the console time defined as a period during which console surgeon is operating at console was 124.4 min. While nephrectomy and preparation of the distal ureter with a period of 36.2 and 48.2 min was performed for all patients, lymphadenectomy was performed for 3 patients and took 30.3 min on average. The second step for dissection of distal ureter was enabled through the afore mentioned re-docking procedure. Re-docking took a mean time of 7 min.

**Table 2:** Timetable of surgery

Surgical steps:	Trocar placement (min)	Time to artery (min)	Nephrectomy (min)	Lymphadenectomy (min)	Re-Docking time (min)	Bladder cuff (min)	Console time (min)	Surgery time (min)
Patient 1	38	9	50	60	7	50	190	270
Patient 2	24	6	38	x	6	36	91	160
Patient 3	35	8	27	15	7	42	122	170
Patient 4	22	4	38	x	7	55	105	165
Patient 5	21	5	28	16	8	58	114	181
Median	24	6	38	16	7	50	114	170
IQR	(22–35)	(5–8)	(28–38)	(15–60)	X	(42–55)	(105–122)	(165–181)
Mean	28	6.4	36.2	30.3	7	48.2	124.4	189.2

IQR: interquartile range

### Complications

Median EBL was 150 ml (Table 1). EAUiaiC showed a single grade 1 intraoperative complication which is referred to change to a 30° camera for management of bladder cuff due to insufficient vision. Postoperative complications using Clavien–Dindo classification showed no complication exceeding grade ≥ 3a or higher. One patient received transfusion of erythrocyte concentrates on day 3 due to serum hemoglobin of 7.9 mg/dl (Clavien grade 2). Mean hospital stay was 5.4d. Patient mobilization as well as bowel movement were on time.

### Histopathology

All five patients suffered from UTUC (Table 1). In three cases, the disease was located in the collecting systems of the kidney and two times in distal ureter. The mean tumor size was 3.02 cm. Final histological examination revealed 2 × pTa (40%), 2 × pT1 (40%) as well as 1 × pT3 (20%). No positive margins were detected. None of **3** patients experienced positive lymph nodes on final pathology.

### Follow-up

The mean follow-up time was 6 months. During this period no patients experienced systematic recurrence. One patient had multifocal evidence of NMIBC (Non-Muscle Invasive Bladder Cancer) within the first follow-up cystoscopy after 6 months and was treated with TUR-B (pTa, low-grade). No patient has been readmitted for emergency or complications.

### Cystography and chemotherapy

According to the EAU Guidelines with strong recommendation for postoperative bladder instillation with a chemotherapeutic agent, all patients with UTUC were treated with intravesical instillation of mitomycin [1] after sufficient cystography before bladder catheter removal.

## Discussion

To date there are no reports on a surgical technique allowing to perform all surgical steps of a robot-assisted NU using only retroperitoneal space. Due to our novel port configuration and ongoing improvements of robotic systems, we create and establish an approach of completely retroperitoneally executed NU with a bladder cuff resection. Notably, our innovative trocar placement integrates two well-established concepts: the robot-assisted retroperitoneal partial nephrectomy [8] and the robot-assisted transperitoneal adrenalectomy [16]. While creation of the retroperitoneal space is based on the principles of the retroperitoneal partial nephrectomy, the arrangements of the ports is adapted to that of the transperitoneal adrenalectomy with placement of trocars in a curved line parallel to the arcus costalis. Due to this modified port arrangement and after simplified re-docking, nephroureterectomy and dissection of the bladder cuff is both possible through the same ports and fully retroperitoneally.

The nephrectomy portion is thereby quite similar to that described by other groups utilizing retroperitoneal access for robot-assisted NU keeping in mind that none of these studies completed surgery under robotic assistance [4, 12, 13]. Compared to the transperitoneal approach which is currently the most common technique, we observed similar surgical characteristics with our technique. Whereas Patel et al. demonstrated a mean surgery time of 152 min [6] and Darwiche et al. of 184 min [8], our operative time was as long as 189.2 min (median 170 min; IQR 165–181). As expected, trocar placement took longer with 28 min (median 24 min; IQR 22–35**)** due to a more sophisticated creation procedure of the retroperitoneal space. Raheem et al. reported a docking time of 17.8 min using DaVinci^R^Xi for transperitoneal partial nephrectomy [17]. Several surgical techniques reported the need of re-docking and relocation of the robot for management of the bladder cuff, what generally leads to an additional operative time of 30–60 min [4]. Due to the possibility of a 180°-twist of the main robotic joint right after finishing nephrectomy portion without the need of relocation, this adds only a negligible additional time of 7 min for our RRNU.

Intra- and postoperative complications were rare. No conversion to open or laparoscopic surgery was observed. While we noted a median EBL of 150 ml (IQR 100–250), several studies described a comparable EBL of 120–200 ml [6–8, 18]. Furthermore, we reported an intraoperative change of procedure in terms of switching from 0° to 30°camera for management of the bladder cuff (EAUiaiC grade 2 complication) what might be attributed to the previous radical prostatectomy in this patient. After switching to a 30°camera bladder cuff dissection was easily possible. In total, nephrectomy portion using four-arm configuration was feasible, while the three-arm configuration for the lower portion led sometimes to clashing of the instruments. Nevertheless, proper dissection of the bladder cuff was possible in all cases, what is of a great importance considering the high recurrence rate of up to 30–64% in cases with incomplete removal [19]. Interestingly, Wu et al. just defined novel criteria for sufficient bladder cuff dissection including en-bloc excision, mucosa-to-mucosa reliable closure and no urine spillage [20].

No major postoperative complications or readmissions after discharge were observed. Interestingly, hospital stay was 5.4 days (range 5–7) while other groups reported of 2–7 days (range 1–14) [6–8, 18]. These varying data may be related to differences in health care systems and department policies impeding a reliable comparison [21].

Attention should be paid here to the potential concern of heat conduction and burning of port site when inserting a standard 8-mm robotic trocar in the 12-mm Hasson trocar often raised by technical professionals and stakeholders. We usually apply this approach due to lower expenditures related to one-way Hasson trocar and retroperitoneal balloon. In the present series as well as in our retroperitoneal partial nephrectomy procedures, we have never encountered any adverse events related to the aforementioned issue. However, unless there is a reliable evidence for the same safety, Hasson cone of Intuitive Surgical appears to be a safer option and should be a preferable choice in the future.

Our successful implementation of RRNU is underpinned by histological and postoperative findings. All patients with UTUC showed no positive surgical margins even if mean tumor size was 3.02 cm. Studies including larger cohorts noted positive margins of 2–23% [6, 7, 18, 22]. For closing the bladder defect, we performed a one-layer closing technique with a barbed suture and intraoperative watertightness testing by irrigating the catheter demonstrated no urine leakage in all cases. In addition, postoperative drain removal was performed between day 2–4 after excluding urine admixture in drain fluid and sufficient cystography demonstrated regular recovery of the bladder defect. Within a follow-up of 2–8 months no patient experienced systematic recurrence while one patient had multifocal localized bladder recurrence of a pTa urothelial carcinoma in the follow-up cystoscopy and a consecutive TUR-B, as is described for 22–47% of all patients with UTUC [1].

Based on the findings from robot-assisted partial nephrectomy, a fully retroperitoneal as compared to a transperitoneal approach might be beneficial for certain aspects. While in some studies it was associated with a reduction in operation time [10, 11, 23], intraoperative EBL [11] and hospital stay [9, 24], major intra- and postoperative complication rates were nearly the same [25]. However minor complications (Clavien grade 1–2) seem to be even less frequent for patients undergoing a retroperitoneal approach [25]. It is noteworthy that in general studies comparing postoperative patient reported outcomes particularly concerning pain and first bowel movement between retroperitoneal versus transperitoneal approach for kidney surgery are yet missing.

Our study has some limitations. We developed a new surgical technique based on observations of a single-center study while surgery was always carried out by the same operation team. In addition, we describe a small cohort and regarding to postoperative follow-up, especially referring to tumor specific long-term observations, we covered only a short period after surgery. Nevertheless, we believe to have created a feasible and generalizable surgical technique that should be compared to other techniques in prospective studies.

## Conclusions

We present the first report on RRNU performed by robot-assistance entirely through a retroperitoneal approach. This innovative surgical technique restricted only to the retroperitoneal space offers the possibility to become standard of care for selected patients.

## Supplementary Information

Below is the link to the electronic supplementary material.

Supplementary file1 (All rights reserved World Journal of Urology and Authors. Only for private use) (PPTX 259517 KB)
